# Studying Function and Behavior in the Fossil Record

**DOI:** 10.1371/journal.pbio.1000321

**Published:** 2010-03-02

**Authors:** Michael J. Benton

**Affiliations:** Department of Earth Sciences, University of Bristol, Bristol, United Kingdom

## Abstract

It is easy to dismiss reconstructed organisms and behaviors from the past as “mere speculation”, but empirical evidence, comparison with modern analogs, and biomechanical modeling can provide remarkable insights.

Inferring the behavior and function of ancient organisms is hard. Some paleontologists would say that it cannot be done because such hypotheses can never be testable, whereas others would say that this is surely a prime task for paleontology—to seek to bring ancient organisms back to life.

These issues have long troubled paleontologists. The founder of comparative anatomy, Georges Cuvier (1769–1832), insisted on the common pattern of the skeleton of living and fossil vertebrates and that anatomy could be reconstructed with confidence from incomplete fossil remains. Further, he argued that the skeleton of a living or extinct animal held unequivocal clues about function and behavior. Cuvier saw his mission to establish rules for comparative anatomy that would allow paleontologists to make certain statement with clarity and confidence [1], a key principle today, what one might call “evidence-based reconstruction” (for example, sharp teeth indicate a diet of meat rather than plants, or mammalian characters in the teeth indicate that the unknown animal was endothermic and nourished its young from mammary glands) as opposed to speculation (“this dinosaur was purple because I guess it was”).

## Form, Function, and Behavior

It can be assumed that biological structures are adapted in some way and that they have evolved to be reasonably efficient at doing something. So, an elephant's trunk has evolved to act as a grasping and sucking organ to allow the huge animal to reach the ground and to gather food and drink. Cuvier realized that form reflected function, even though he interpreted such exquisite adaptations as evidence for design rather than evolution. But we must be cautious of over-interpretation, perhaps assuming that everything is an adaptation and that adaptations are all perfect [2].

Fossils can provide a great deal of evidence about function. For example, the hard skeleton of a fossil arthropod reveals the number and shape of the limbs, the nature of each joint in each limb, perhaps also the mouthparts, and other structures relating to locomotion and feeding. Exceptionally preserved fossils may reveal additional structures, such as the outline of the tentacles of a belemnite or ammonite, hair and feathers, and muscle tissue or sensory organs. In vertebrates, there may be muscle scars on the surface of the bone and particular knobs and ridges (processes) that show where the muscles attached and how big they were. In addition, the maximum amount of rotation and hinging at each joint in a skeleton can be assessed because this depends on the shapes of the ends of the limb bones. Such practical observations can at least limit the options; as an example, in the debate over whether pterosaurs could walk with the limbs tucked right beneath the body (parasagittal posture) or sprawling in cowboy posture to the sides, the fossils showed that the latter was the case [3].

There are three approaches to inferring function and behavior from fossils—empirical evidence, comparison with modern analogs, and biomechanical modeling.

## Empirical Evidence

Paleontologists are inquisitive by nature, and they gather evidence of all kinds to test their hypotheses. Evidence about the lifestyle of an ancient plant or animal may come from the enclosing rocks, associated fossil remains, associated trace fossils, and particular features of the body fossils themselves. The rocks can give clear evidence about ancient climates, and associated fossils indicate possible prey and predator relationships.

Trace fossils, such as tracks and burrows, can sometimes be linked with their makers, and then used to look at modes of locomotion and whether animals burrowed or not [4],[5]. Tracks also give surer evidence about some aspects of locomotion than the bones themselves. For example, manipulating bones can allow a paleontologist to work out whether a tetrapod stood upright like a mammal or held its arms and legs sideways in a sprawling posture. But it's not always easy. Footprints show precisely whether the feet fell in a single line or were far apart, and a study across the famous end-Permian mass extinction 252 million years ago shows a dramatic shift from sprawling to upright posture among virtually all tetrapods at the same time [6].

Fossil dung or stomach contents can often be associated with the producer, and paleobiologists who are so inclined can tease apart fossil dung under the microscope and determine the key constituents of diet. A famous 44-cm–long coprolite dropped by *T. rex* contains pulverized bones of ornithischian dinosaurs that had been corroded to some extent by stomach acids, but not entirely destroyed [7]. This suggests a relatively rapid transit of food material through the gut. The teeth of ancient animals can indicate diet, and detailed study of teeth of fossil mammals can even indicate the kinds of plants they were eating, based on fine scratches and grooves seen under the microscope [8].

**Figure 1:**
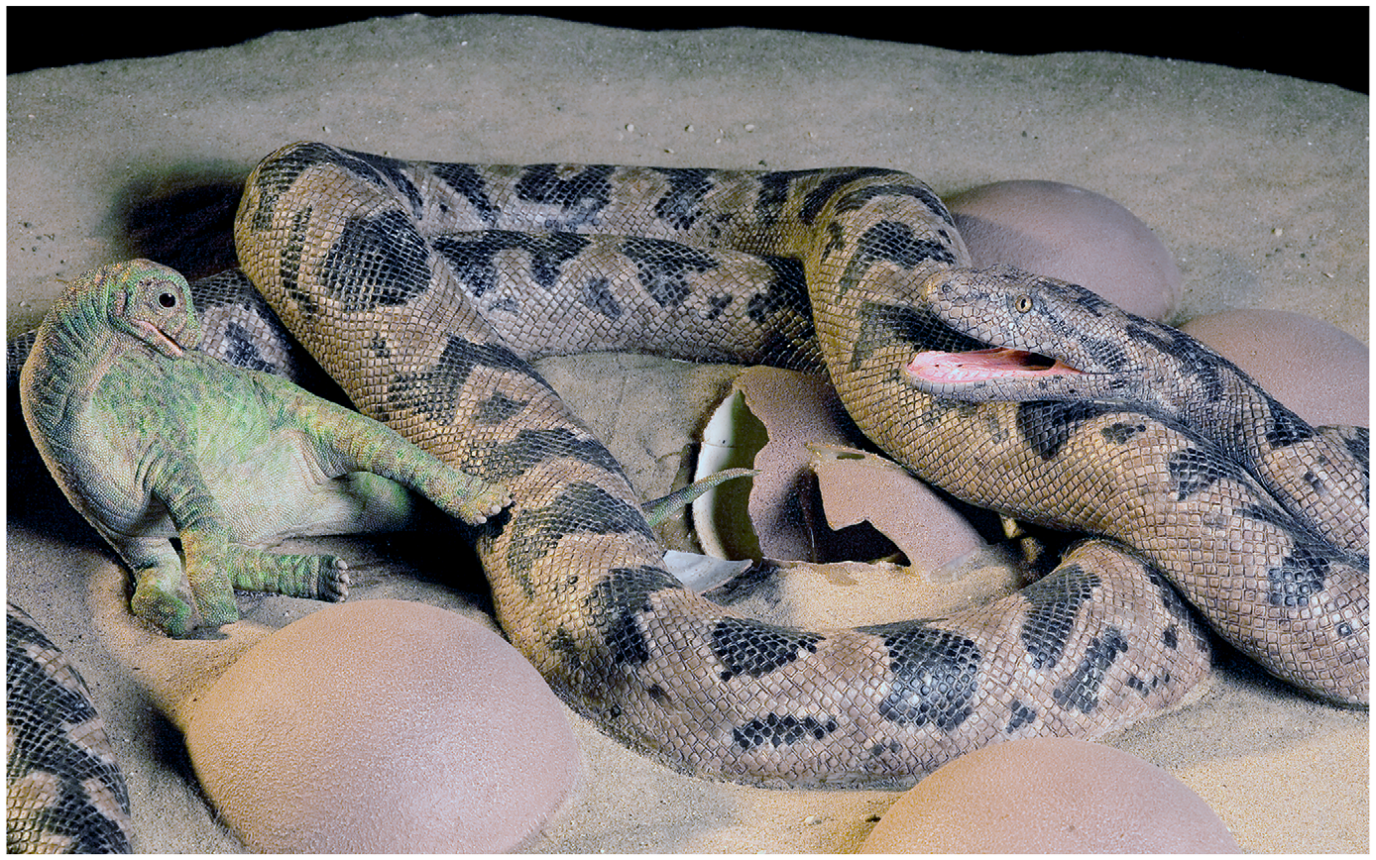
A Cretaceous snake feeding on hatchling sauropod dinosaurs. A 3–5-meter-long madtsoiid snake, *Sanajeh indicus*, waits to feed on hatchling sauropod dinosaurs as they emerge from their eggs, in a scene from the Upper Cretaceous, some 70 million years ago. The sculpture is based on a fossil dinosaur nest from western India, reported in this issue of *PLoS Biology* [11]. The scales and patterning of the snake's skin is based on modern macrostomatan snakes, relatives of the fossil form. The hatchling dinosaur is reconstructed from known skeletal materials, but its color is conjectural. The eggs are based directly on the fossils. In making their detailed paleobiological interpretations, Wilson and colleagues [11] used all three methods advocated in this review—empirical observations of a remarkable specimen, coupled with comparison with modern analogs and biomechanical modeling. In detail, the authors incorporated museum-based research, field research, stratigraphy and sedimentology, histology, embryology, and use of modern analogs into their interpretation of *Sanajeh*. (Sculpture by Tyler Keillor and original photography by Ximena Erickson; image modified by Bonnie Miljour).

Sometimes one organism is preserved *in flagrante delicto*, as it were, feeding upon another—for example, small leaf-eating insects within fossil plant stems [9], or a fish that died choking on a fish it was trying to swallow [10]. Jeff Wilson and colleagues report another such remarkable specimen in this issue of *PLoS Biology*, a snake preserved complete and wrapped around a crushed dinosaur egg in a nest of otherwise unbroken eggs, from the Late Cretaceous of India [11]. It seems most likely, as the authors argue, that this 3.5-meter–long snake was waiting and snatching juveniles as they hatched (see Figure 1). Of course, we cannot be entirely sure unless further specimens come to light showing the bones of juvenile dinosaurs in the stomach region of the snake. In this case, and others, the specimens are key, and the care taken by their collectors and investigators to extract every fine detail.

In all cases, these are not “mere observations” or descriptions of rather obvious data, but rather the statements are hypotheses like any other, subject to refutation at any time. The Cuvierian example noted earlier, that a jaw that bears mammalian teeth can tell the paleontologist that it came from, say, a marsupial mole of a particular family, and the needle-like teeth indicate that it fed on insects, is based entirely within the hypothetico-deductive model. Just as the classic assertion that “all swans are white” was refuted by the discovery of the black Tasmanian swan, each of the assertions/claims made by the paleontologist is open to close inspection and refutation based on new evidence.

## Comparison with Modern Analogs

It is probable that function and behavior of a fossil bat should be inferred from comparisons with living bats. But should a dinosaur be compared with living relatives (e.g., birds or crocodiles) or with living animals with apparently similar function (e.g., elephants or rhinos)? Phylogeny might be thought to trump general similarity, but does it? Perhaps it would be pointless to compare a *Diplodocus* with a sparrow—their body size, morphology, and presumed modes of life are wildly different.

But something informative does come from phylogeny. At one level, parsimony allows paleobiologists to infer the presence of soft-tissue characters and behaviors. A development of the parsimony principle is the *extant phylogenetic bracket* (EPB) [12]. According to this principle, osteological correlates of unpreserved features are identified, and these allow inferences about the presence of unpreserved features. At a simple level, we could say that *Tyrannosaurus rex* presumably had an eyeball with certain properties, because its bracketing living relatives—birds and crocodiles—share many common characters in their eyes. A further example, perhaps a little more impressive, is the prediction that fossil eggs will some day be found in the Carboniferous. The reasoning is that all living amniotes (i.e., reptiles, birds, and mammals) lay hard-shelled eggs, even though egg laying has been replaced by live birth in most mammals and some snakes and lizards. Thus, the first amniote in the Carboniferous, over 300 million years ago, presumably laid a hard-shelled egg, even though the oldest fossil eggs are known only from the Triassic, 100 million years later [13].

Parsimony and the EPB are now widely used in discourse about the remarkable feathered birds and dinosaurs from the Jehol Group of China (Early Cretaceous, 131-120 Ma). When specimens of the small theropod *Sinosauropteryx* were announced [14] with simple filament-like feathers, paleontologists looked at the phylogenetic trees and realized that this took the origin of feathers back to the base of the Middle Jurassic, some 175 million years ago. This is because *Sinosauropteryx* is a basal coelurosaur, and the first coelurosaurs are known from the base of the Middle Jurassic; the most parsimonious assumption is that all coelurosaurs possessed some kind of feathers from the start. Note that the *Sinosauropteryx* filaments are debated, and some [15] argue they are not feathers but connective tissue, but close study suggests otherwise [16]. In any case, feathers have been reported from nearly every other coelurosaur lineage, and so their origin deep within the phylogeny of theropod dinosaurs appears assured. Knowing the arrangement of feathers, and perhaps their colors and patterns [16]–[18], may allow paleobiologists soon to speculate, rationally and calmly, of course, about whether certain dinosaurs used their brightly colored and patterned feathers for camouflage, warning, sexual display, establishing pecking order, or other behaviors and functions.

## Biomechanical Modeling

**Figure 2:**
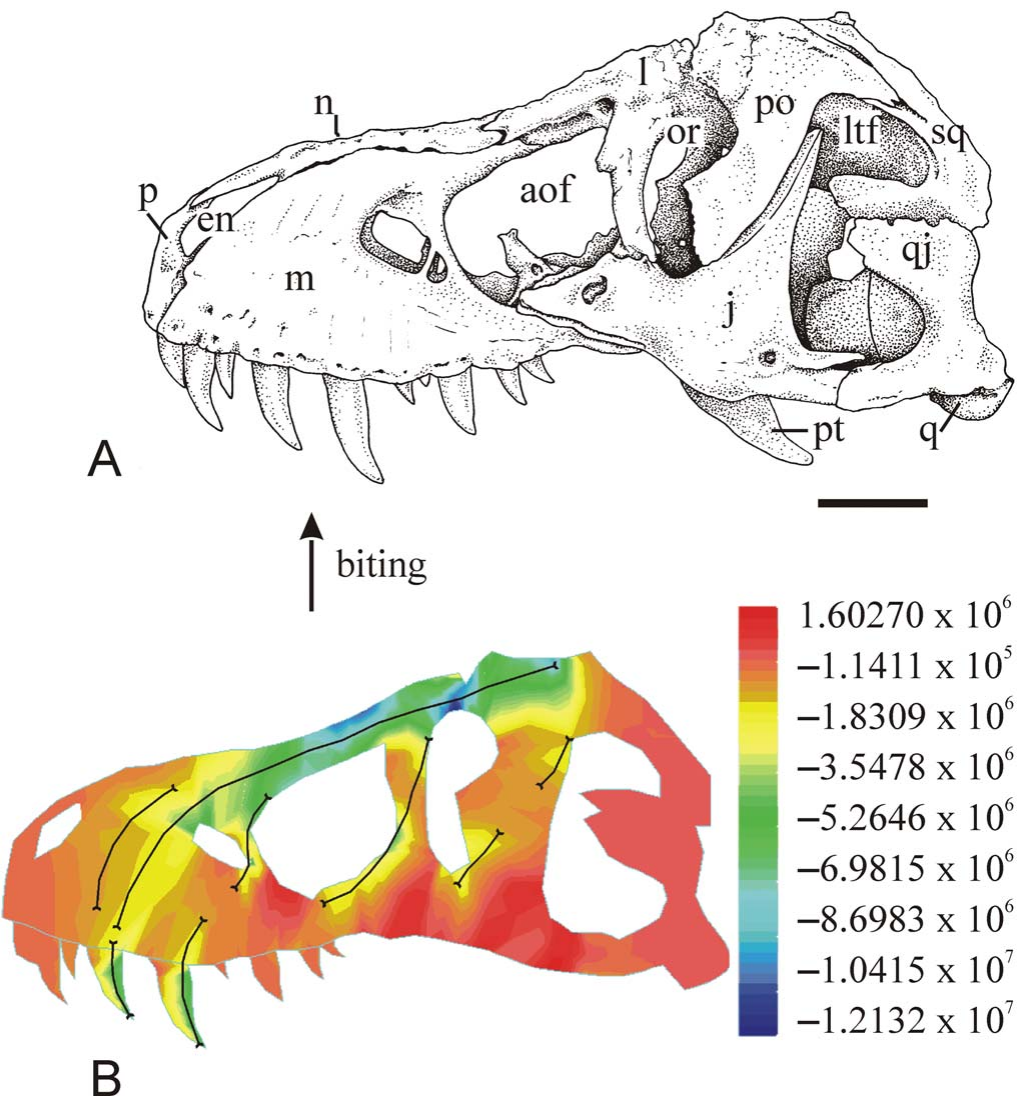
Finite element analysis of the skull of *T. rex*. The skull of *T. rex* is perhaps one of the most talked about fossils of all time, coming as it does from perhaps the most fearsome, and certainly the largest, terrestrial predator that ever lived. But the anatomy of the skull reveals a paradox; while *T. rex* is assumed to have been capable of producing extremely powerful bite forces, the skull bones are quite loosely articulated. Does this mean that the skull would have expanded and distorted if its owner bit too hard into a *Triceratops* carcass, or did *T. rex* have to control its bloodthirsty efforts? Emily Rayfield [19] studied all the available skulls (A) and constructed a mesh of triangular elements, small triangular or cuboid cells that define the 3-D shape in preparation for engineering analysis. The technique used is FEA, a numerical method worked out in the 1940s to study the physical properties of buildings. In Rayfield's FEA model of the *T. rex* skull, modeled bite forces of 31,000–78,060 newtons were applied to individual teeth, and the distortion of the element mesh observed (B). The bite forces had been taken from calculations by other paleobiologists, and from observations of tooth puncture marks (a piece of bone bitten by *T. rex* showed the tooth had penetrated the bone to a depth of 11.5 mm, equivalent to a force of 13,400 newtons, or about one-and-a-half tons). Rayfield's results show that the skull is equally adapted to resist biting or tearing forces and therefore the classic “puncture-pull” feeding hypothesis, in which *T. rex* bites into flesh and tears back, is well supported. Major stresses of biting acted through the pillar-like parts of the skull and the nasal bones on top of the snout, and the loose connections between the bones in the cheek region allowed small movements during the bite, acting as “shock absorbers” to protect other skull structures. (Image Credit: Emily Rayfield)

Biomechanical models, combined with considerations of modern analogs, provide powerful insight into certain aspects of the moving parts and skeletons of ancient organisms. Opportunities have been hugely expanded by the relative ease with which 3-D structures, such as shells, bones, and skeletons, may be scanned and imaged. These images may then be tested using standard engineering software to determine how the structure was shaped by stresses and strains of walking, running, feeding, or head butting. A useful modeling approach is finite element analysis (FEA) [19],[20], a well-established method used by engineers to assess the strength of bridges and buildings before they are built, and now applied to dinosaur skulls (see Figure 2), among other fossil problems.

A number of attempts have been made to understand how dinosaurs walked and ran, and of course everyone focuses on *T. rex*. It is reasonable to assume that the laws of physics and the principles of biomechanics were the same in the past as they are now. For example, the starting point in studying the locomotion of any animal, especially a biped, is to establish the center of mass—whatever happens, the animal must not fall over. The center of mass for a living or extinct animal can be determined either from solid models or from calculations of the distribution of tissues and air spaces through slices of the 3-D restored body [21],[22]. In *T. rex*, the center of mass lay just in front of the hips, and the tail balanced the body over the hips that acted as a fulcrum, giving a most natural stance with the backbone held almost horizontal. This is a major improvement over the old-style kangaroo poses that people used to use for dinosaurs, where the animal's body was more vertical than horizontal and the tail rested on the ground. This is only the beginning, however, and *T. rex* could be imagined walking and running in a variety of poses [23],[24].

**Figure 3:**
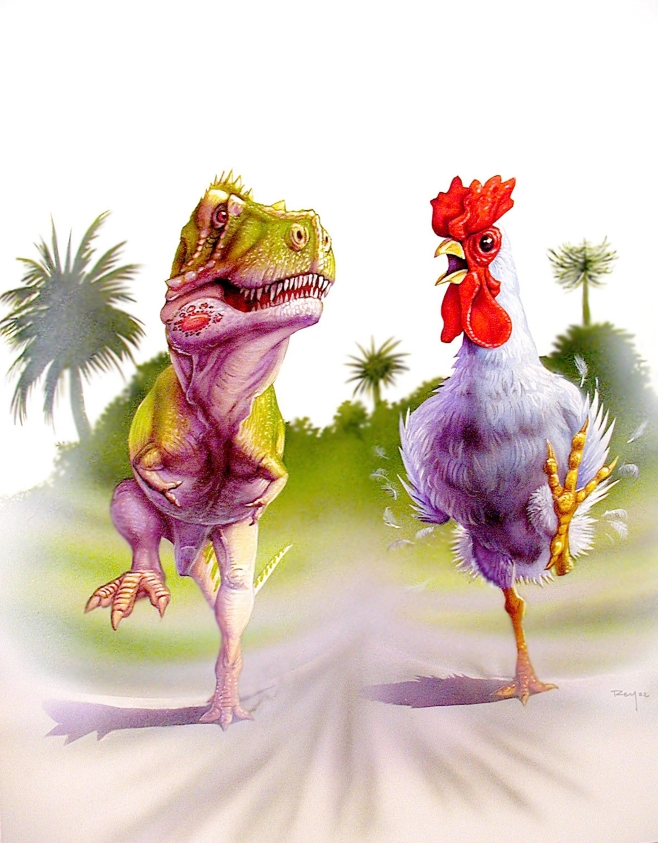
*T. rex* trotting along beside a *T. rex*-sized chicken. Calculations of the muscle mass required to power a fast-running *T. rex* showed that this was impossible—a 6-tonne chicken would have needed leg muscles making up almost 100% of its body mass. Realistically, *T. rex* had the muscles to run at about 5 meters per second (18 km/h, 11 mph) [26]. (Painting courtesy of Luis Rey.)

But how fast could *T. rex* run? Here, many estimates have been made, ranging from a speedy 20 meters per second (72 km/h, 42 mph) to a more sedate 5 meters per second (18 km/h, 11 mph), the speed of a human long-distance runner. Many approaches were used, and these illustrate the ingenuity of paleobiologists. For example, fossil trackways can indicate speeds: there is a constant relationship between the spacing of footprints (stride length), leg length, and speed [25]. Others made calculations based on relative lengths of the leg bones, or on assumptions about the risk of injury if the animal fell, or by using calculations of stress and strain (the faster you run, the greater the impact as the foot hits the ground). Most recently, the question has been resolved by simple calculations based on estimated leg muscle volume; the major leg muscles that power the stride are proportional to body mass and speed [26]. At speeds faster than 5 meters per second, the 6-tonne *T. rex* would have needed leg muscles that were proportional to those of a chicken (see Figure 3) and at 20 meters per second, the highest speed previously assumed, the leg-powering muscles would have made up to 86% of total body mass. In a further set of calculations, Pontzer and collegues [27] show that the biomechanics of running and metabolic rate are intimately linked, and, based on evidence from extant tetrapods, they can identify that the larger dinosaurs at least exceeded the maximum aerobic capabilities of modern ectotherms. This means they were functionally endothermic, although this may well have arisen through inertial homeothermy because of their large size.

Paleobiologists then need not make wild guesses in reconstruction of behavior and function in the past. Ingenious interpretations and spectacular discoveries, such as the snake on the dinosaur nest [11], can sometimes give us remarkable insights into the long-lost life of the past.

## Abbreviations

extant phylogenetic bracket (EPB)
finite element analysis (FEA)
